# Reverse Lymphatic Flow in Lower Extremity Lymphedema Visualized on Single-Photon Emission Computed Tomography—A “Downflow Effect”

**DOI:** 10.3390/jcm15030942

**Published:** 2026-01-23

**Authors:** Jun Won Lee, Han-Sang Song, Chulhan Kim, Tae-Yul Lee, Hi-Jin You, Deok-Woo Kim

**Affiliations:** 1 Department of Plastic and Reconstructive Surgery, Kangnam Sacred Heart Hospital, College of Medicine, Hallym University, Seoul 07441, Republic of Korea; ljw2023@hallym.or.kr; 2 Department of Plastic Surgery, College of Medicine, Korea University, Seoul 02841, Republic of Korea; sssonggum@kumc.or.kr (H.-S.S.); tylee0919@korea.ac.kr (T.-Y.L.); u2jin2@korea.ac.kr (H.-J.Y.); 3 Institute of Advanced Regeneration and Reconstruction, College of Medicine, Korea University, Seoul 02841, Republic of Korea; 4 College of Medicine, Chungbuk National University, Cheongju 28644, Republic of Korea; chulhankim@chungbuk.ac.kr; 5 Department of Nuclear Medicine, Chungbuk National University Hospital, Cheongju 28644, Republic of Korea

**Keywords:** lymphedema, lymphatic system, lymphography

## Abstract

**Background:** Patients who undergo pelvic lymphadenectomy for gynecologic or genitourinary cancers have an increased risk of developing lower extremity lymphedema. Although total lymphadenectomy is performed, bilateral lower extremity lymphedema is rare. A state-of-the-art radiologic technique, single-photon emission computed tomography (SPECT) with radioisotope injection, was used to establish lymph flow physiology and identify retrograde lymphatic flow in patients with lower extremity lymphedema after lymphadenectomy. **Methods:** Data from patients who underwent treatment for lower extremity lymphedema were collected from January 2017 to December 2018. These patients had gynecological or genitourinary cancers and had undergone pelvic lymphadenectomy. Among them, 10 were evaluated for reverse lymph flow using SPECT. The radioisotope was injected solely into the subdermal area of the healthy foot, not the affected foot, in contrast to other studies. Four hours later, SPECT images were obtained and analyzed. The radiologic results were correlated with clinical observations. **Results:** Most patients had undergone surgery for gynecological cancers. The mean disease duration was 9.4 ± 8.1 years. Retention in the pelvis and hip was confirmed in seven out of ten patients; six patients showed reverse lymphatic flow in the affected limb. **Conclusions:** SPECT-CT imaging after tracer injection into the unaffected limb revealed retrograde lymphatic flow toward the clinically affected side in a substantial proportion of patients with unilateral lower-extremity lymphedema.

## 1. Introduction

Lymphedema is a progressive disease that exhibits swelling and fibrosis in areas where lymphatic flow has been damaged for various reasons. Primary lymphedema is a condition caused by hereditary or genetic abnormalities that result in malformation of the lymphatic system. Secondary lymphedema refers to lymphedema arising as a result of disease or iatrogenic causes such as surgical intervention. Cancer resection is one of the main causes of secondary lymphedema; the incidence of secondary lymphedema has increased with the increase in number of patients suffering from cancers.

The pathogenesis of this disease remains unclear. The pathophysiology of the primary and secondary lymphedema is not significantly different [1]. The initial lymphatic injury may lead to the accumulation of lymphatic fluid, which results in adipogenesis and inflammatory processes that initiate the molecular cascade of lymphatic dysfunction. Along with release of inflammatory cytokines, tissue fibrosis, decreased lymphatic pumping, and impaired collateral lymphatic formation occur during the process, leading to a vicious cycle of further fluid stasis and activation of the inflammatory response [2].

Secondary lymphedema can arise from various factors such as infections, radiations, trauma, and surgeries. Surgery-induced lymphedema specifically results from the removal of the lymph node basin, disrupting lymphatic flow and drainage. Inflammatory cells, including CD4 T cells, Tregs, macrophages, and dendritic cells, contribute to the pathophysiology by triggering an inflammatory response, causing lymphatic system dysfunction, promoting collateral lymphatic growth, impairing immune response, and affecting lymphatic vessel contraction [3].

According to a meta-analysis, the incidence of lower extremity lymphedema after the treatment of gynecologic or genitourinary tumors is as high as 20% and 10%, respectively [4].

While lymphatic tissue on both sides is resected during pelvic lymphadenectomy, the incidence of bilateral lower extremity lymphedema is uncommon [5]. Bilateral involvement is seen in 16.1% of cancer-related lymphedema and 61.0% in noncancer-related lymphedema [6].

Studies on the pathophysiology of this phenomenon are lacking, and current studies on lymphatic flow include administration of a contrast agent in the affected limb. We hypothesized that the damaged pelvic lymphatic structures may lead to the retention of lymph fluid, which literally “falls down” (flows in reverse) to the leg with weaker lymphatic structures than the others.

Although various modalities exist to trace lymph flow in vivo, lymphoscintigraphy is considered the most definitive diagnostic tool. Several methods, including indocyanine green (ICG) lymphangiography, single-photon emission computed tomography (SPECT), and magnetic resonance (MR) lymphography, are used for diagnosis [7].

SPECT is a relatively new technique for visualizing the physiological changes and flow of lymphatic fluid. It is used for sentinel lymph node mapping and visualization of lymphatic structures. The relationship between radioactive signals and other tissues can be readily seen on 3D-reconstructed images [7].

In this study, we propose a less common concept for lymph flow in lower-extremity lymphedema as a “downflow” from the lymphatic reserve (pelvic retention) to the damaged water pipe (flowing in reverse into the affected limb) using SPECT imaging.

## 2. Materials and Methods

### 2.1. Patient Selection

The study was conducted in accordance with the principles of the Helsinki Declaration (2013 revision), after obtaining approval from the Institutional Review Board (IRB No. 2022AS0255; 6 October 2022). Informed consent was obtained from all participants for the SPECT/CT scan. The medical records of patients with advanced-stage secondary lymphedema in the lower extremities between January 2017 and December 2018 were analyzed. Reverse lymph flow was evaluated using SPECT. The data were further classified in two groups: reverse and non-reverse.

### 2.2. Radiological Visualization of the Lymphatic Flow (SPECT/CT)

A technetium-99m–labeled stannous phytate radiotracer was used for lymphatic imaging, with a total administered activity of 3–5 mCi. The tracer was prepared in three separate 1-cc syringes, each containing approximately 1–2 mCi diluted to an injectable volume of 0.1–0.3 cc. Injections were performed sequentially into three distinct interdigital web spaces of the clinically unaffected foot: between the first and second toes, the second and third toes, and the fourth and fifth toes. We did not inject the radioisotope into the lymphedematous limb, marking a key difference from prior studies [8]. All injections were administered consecutively during a single session, ensuring distribution across multiple lymphatic entry points rather than repeated injection at a single site. After tracer injection, patients were not required to remain in the nuclear medicine department and were allowed to engage in usual daily activities without specific restrictions. Delayed imaging was performed 4 h after tracer administration.

All examinations were conducted using a Siemens Symbia Intevo 6 hybrid SPECT/CT system (Siemens Healthineers, Forchheim, Germany). CT acquisition was performed at 130 kVp with automatic mAs modulation. SPECT acquisition parameters included a 256 × 256 matrix, a low-energy high-resolution (LEHR) collimator, 30 projections over 360°, and an acquisition time of 40 s per projection, with an energy window centered at 140 keV and a 15% window width. Image reconstruction was performed using Flash 3D (Siemens Healthineers), an iterative reconstruction algorithm with resolution recovery, selected to accommodate low count statistics associated with subcutaneous lymphatic tracer injection. Reconstruction parameters included a pixel size of 2.40 mm × 2.40 mm, smoothing of 10.0 mm, 24 iterations, and 2 subsets.

The images were processed to obtain higher saturation images to clearly identify the lymphatic flow in the pelvic space and affected lower extremity. The radiological results were correlated with clinical observations, including size, volume measurement, and clinical images.

### 2.3. Data Collection

Basic demographic data, including sex, age, disease duration, ISL stage, medical history, and body mass index (BMI), were collected (Table 1). The size and volume of the lower extremities were measured and clinical photographs were taken. Disease duration was defined as the interval from the first presentation of lymphedema-related symptoms to the date of SPECT/CT imaging and was applied consistently to both primary and secondary lymphedema cases. The SPECT data included the presence of pelvic retention or reverse flow to the affected limb, as well as the level of signs of lymphedema, including dermal diffusion. Lymphedema life impact scale (LLIS) was administered to all the patients [9]. Limb circumference was measured at six points: (1) upper thigh, (2) midthigh, (3) superior border of the patella, (4) inferior border of the patella, (5) midcalf, and (6) ankle. Subsequently, the circumference ratios of both limbs were calculated at each point using the following formula: (circumference of the affected limb)/(circumference of the unaffected limb).

### 2.4. Measurement of Limb Volume

The volume of each limb was calculated according to the previously described formula for a truncated cone [10]. The circumferences measured at points 2 and 5 were used to calculate the volume. The volume ratio of both limbs was calculated using the following formula: (volume of the affected limb)/(volume of the unaffected limb).

### 2.5. Statistical Analysis

Demographic data and results of the two patient groups were compared using Fisher’s exact and independent *t*-tests. Statistical analyses were performed using IBM SPSS Statistics 22.0 (IBM Corp., Armonk, NY, USA). *p*-value under 0.05 was considered statistically significant.

## 3. Results

Ten patients were included in the study, with a mean patient age of 53.1 years. Eight patients had ISL stage 2 (80.0%), while two patients had ISL stage 3 (20.0%). Two patients had primary lymphedema, whereas eight patients had secondary lymphedema after pelvic node dissection (20.0% and 80.0%, respectively). Cervical cancer was the predominant cause (70.0%) for lymphedema; only one patient had endometrial cancer (10.0%). Six patients (60.0%) had no medical history other than cancer. All the patients received chemotherapy (100%) and radiotherapy (100%). None had clinically evident bilateral lymphedema; six patients had right-sided involvement, and four had left-sided involvement. All patients received conservative therapy such as compression, and the mean duration of the disease was 9.4 years.

Among the 10 patients included in the study, eight patients had secondary lower-extremity lymphedema following cancer treatment, while two patients had primary lymphedema without any history of malignancy.

In the primary lymphedema subgroup, heterogeneity in lymphatic flow patterns was observed. One patient demonstrated retrograde lymphatic flow extending from the unaffected limb to the upper thigh level of the affected side, whereas the other patient did not exhibit retrograde flow on SPECT/CT imaging. The patient with retrograde lymphatic flow had a long disease duration of 30 years and was classified as ISL stage 3, whereas the patient without retrograde flow had a disease duration of 10 years and ISL stage 2 lymphedema.

The patient demographics and disease characteristics are summarized in Table 1 and Table 2, respectively.

Radiological analysis showed that one patient had only pelvic retention on the contralateral side (10.0%), while most cases had pelvic retention on the side presenting reverse lymph flow (60.0%). The extent of reverse flow was limited to the thigh in all patients. Three patients did not have any findings (neither retention nor reverse flow) (Table 3).

There were seven patients in the reverse group and three in the non-reverse group. There was no statistically significant difference in the mean circumference ratio of the affected to unaffected limb, at all six points, between the two groups. Furthermore, the LLIS scores of the two groups did not differ in the three domains (physical, psychological, and functional concerns) or the total scores (Table 4).

## 4. Discussion

Although numerous attempts to establish the pathophysiology of lymphatic flow and observational findings have been made, associations among impaired lymphatic drainage, inflammation, adipose tissue accumulation, and fibrosis are not well understood [11].

Several imaging studies have shown subcutaneous or intravascular lymphostasis, dermal backflow, dilated lymphatic vessels on the affected side, which is consistent with our findings [12,13,14,15,16,17,18].

While these studies injected the isotope on both sides or into the affected extremity, our study provides a unique perspective by injecting the isotope only into the unaffected foot. We believe that this provides a better understanding of the interconnected characteristics of the lymphatic flow and relationship between each lymphatic structure (the healthy limb, pelvis, and the affected limb).

Understanding lymphatic flow to the pelvis is challenging when the isotope is injected into the affected limb due to the blockage caused by the already damaged lymphatic system. In contrast, although subclinical lymphedema may be present in the healthy limb, lymphatic flow can more readily reach and accumulate in the pelvic nodes [19]. The “downflow effect” may represent one of several factors associated with the observed disease features and unilateral predominance of lymphedema following bilateral pelvic lymph node dissection.

**Figure 1:**
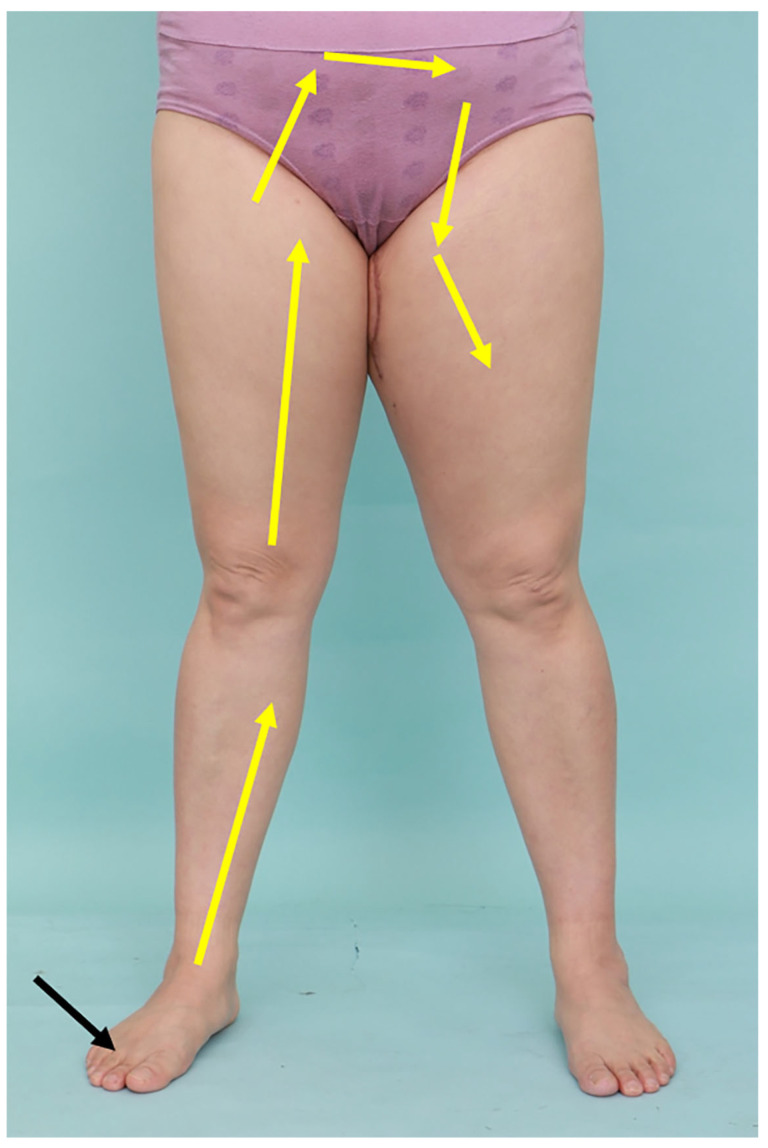
Illustration of the hypothesis that fluid originating from the unaffected leg may flow into the unaffected leg (yellow arrows). This was tested by injecting radioisotope into the healthy foot (black arrow) and using SPECT-CT to assess the distribution of radiotracer four hours after injection.

**Figure 2:**
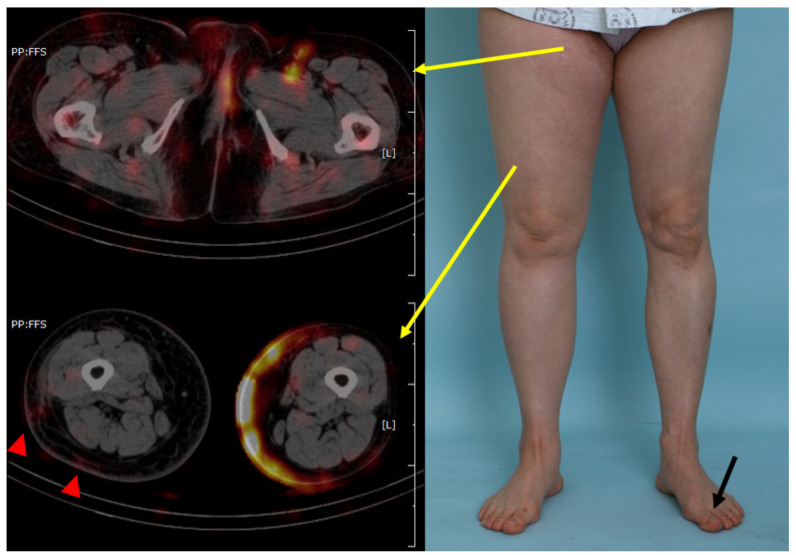
Comparison of the clinical photograph and SPECT scan images of a 55-year-old woman with secondary right-sided lower-extremity lymphedema following cervical cancer treatment with pelvic lymph node dissection. The patient had a disease duration of 9 years. The red area denotes the isotope distribution in the tissue. Radiological findings clearly demonstrate pelvic retention and reverse lymph flow. Yellow arrows indicate the corresponding locations of the SPECT-CT image slices, with the arrow starting points marking the slice level. Red arrowheads denote areas of dermal diffusion, while the radioisotope was injected only into the healthy foot (black arrow).

Excision of the pelvic lymph nodes results in lymphatic retention, which flows down the side of one limb, where the lymphatic structure is weakened or by chance (Figure 1 and Figure 2). The affected side undergoes irreversible impairment of lymphatic function and initiates a vicious cycle of lymphedema, thereby affecting one side more than another.

**Figure 3:**
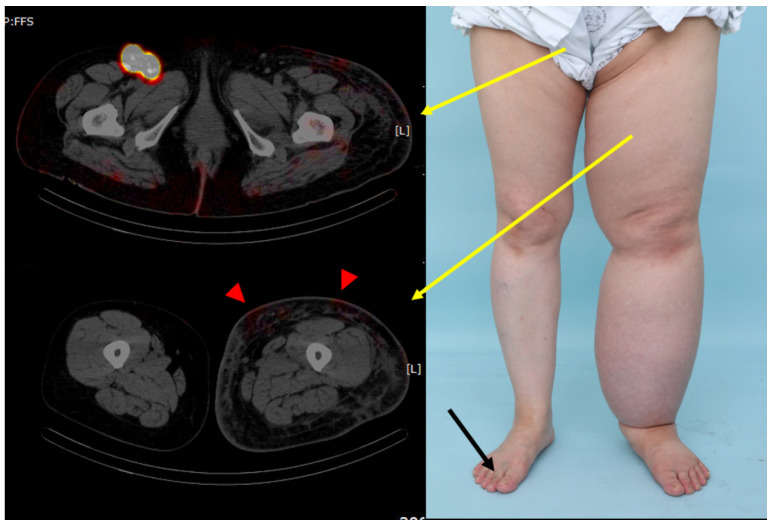
Clinical and radiological images of a patient from the reverse group. The patient was a 50-year-old woman with secondary left-sided lower-extremity lymphedema following treatment for cervical cancer. The patient had a disease duration of 8 years. The red area denotes the isotope distribution in the tissue. Radiological findings suggest pelvic retention and reverse flow of the lymphatics. Yellow arrows indicate the corresponding locations of the SPECT-CT image slices, with the arrow starting points marking the slice level. Red arrowheads denote areas of dermal diffusion, while the radioisotope was injected only into the healthy foot (black arrow).

**Figure 4:**
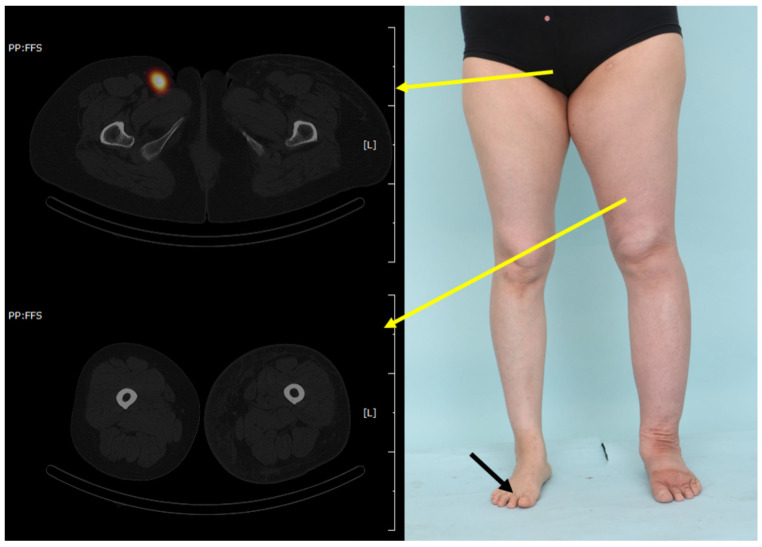
Clinical and radiological images of a patient from the non-reverse group. This patient was a 44-year-old woman with secondary left-sided lower-extremity lymphedema after cervical cancer treatment. The patient had a disease duration of 10 years. A focal area of increased tracer uptake corresponding to a lymph node is observed on fused SPECT/CT imaging. There is no pelvic retention nor reverse lymph flow. Yellow arrows indicate the corresponding locations of the SPECT-CT image slices, with the arrow starting points marking the slice level, while the radioisotope was injected only into the healthy foot (black arrow).

Since there was no statistical difference in the clinical observations between the reverse and non-reverse groups, this theory may not be the predominant cause of lower-extremity lymphedema. The limb circumference and volume ratios between the affected and unaffected limbs, and LLIS scores did not correspond with the radiological results (Figure 3 and Figure 4). Although the clinical observations did not closely correspond with the radiological evidence, this hypothesis need not be completely disregarded as lymphatic retention and reverse lymph flow were observed in most patients. This study only had ten subjects. Thus, studies with larger sample sizes could generate statistically significant results. It is postulated that this “downflow” effect may partially contribute to the pathophysiology of lower-extremity lymphedema.

Lymphatic microsurgical procedures, such as lymphovenous anastomosis (LVA) and vascularized lymph node transfer (VLNT), have been increasingly applied for the treatment of extremity lymphedema. These techniques aim to restore or bypass impaired lymphatic pathways and have been shown to improve limb volume, symptoms, and quality of life in selected patients [20]. However, their effects should be understood in terms of functional drainage improvement rather than reversal of a single upstream lymphatic mechanism. VLNT to the more distal part of the limb (patella, ankle, elbow, and wrist) may not reduce pelvic retention and reverse lymph flow. Preventive pressure garments and devices after lymph node dissection may be advantageous because they block the initiation of the “downflow” effect [21].

The small sample size used to compare the true effects of retention and reverse flow was a limitation of this study. Furthermore, detailed information regarding asymmetry of surgical extent, nodal yield, tumor laterality, and adjuvant treatment intensity was not consistently available in the medical records and could not be systematically evaluated due to the retrospective nature. Although this study was significantly underpowered, a large proportion of the patients showed obvious signs of retention and reverse flow, which supported the hypothesis of “downflow” from the contralateral side of the pelvis.

In addition, patient activity during the uptake period was not standardized. Differences in ambulation or physical activity may have influenced tracer migration and contributed to inter-individual variability in imaging findings.

Due to patients’ socioeconomic status and concerns about excessive radiation exposure, whole-body scans could not be performed. While dynamic lymphatic flow and imaging above the umbilicus would have been beneficial, we believe that pelvic scans are sufficient since the main region of interest was the pelvis. Thus, a whole-body scan was not deemed necessary to validate the hypothesis.

Although shorter observation times or serial dynamic imaging would be advantageous, we limited our approach to delayed imaging to minimize radiation exposure. Despite the limitations of delayed images in showing dynamic flow and lymph node physiology, we were able to observe the downflow.

Dynamic imaging, a larger sample size, and correlation with the ICG lymphography findings could be valuable for further development of this concept in future studies.

## 5. Conclusions

This study demonstrates the presence of retrograde lymphatic flow from the unaffected limb toward the affected side on SPECT-CT imaging in patients with unilateral lower extremity lymphedema. The observed “downflow effect” represents an imaging phenomenon associated with unilateral disease predominance. However, the present findings do not establish causality, and the relationship between lymphatic flow patterns and disease development remains to be clarified. Further prospective studies are required to determine the clinical and mechanistic significance of these observations.

## Figures and Tables

**Table 1:** Patient demographics.

Characteristic	
Sex (Male:Female)	1:9
Mean age ± SD, years	53.1 ± 10.9
Smoking status, n (%)	
None	10 (100%)
Current	0
Medical history other than lymphedema-related disease, n(%)	
None	6 (60.0%)
Hypertension	2 (20.0%)
Diabetes	0
Hepatitis	2 (20.0%)
Others	1 (10.0%)
Mean BMI ± SD, kg/m^2^	26.2 ± 2.9
ISL Stage, n (%)	
Stage 2	8 (80.0%)
Stage 3	2 (20.0%)
Affected side (Right:Left)	6:4
Etiology, n (%)	
Primary lymphedema	2 (20.0%)
Cervical cancer	7 (70.0%)
Others	1 (10.0%)
Lymphatic structure invasion, n(%)	
Pelvic lymph node dissection	10 (100%)
Chemotherapy	10 (100%)
Radiotherapy	10 (100%)
Mean duration of the disease ± SD, years	9.4 ± 8.1
Prior therapies for lymphedema, n(%)	
Compression therapy	10 (100%)
Surgical intervention	0

BMI, body mass index; SD, standard deviation.

**Table 2:** Disease characteristics.

Characteristics	
Circumference ratio (affected to unaffected limbs), mean ± SD	
Upper thigh (point 1)	1.13 ± 0.09
Midthigh (point 2)	1.18 ± 0.11
Superior border of patella (point 3)	1.15 ± 0.10
Inferior border of patella (point 4)	1.19 ± 0.12
Midcalf (point 5)	1.16 ± 0.10
Ankle (point 6)	1.19 ± 0.16
Mean volume ratio (affected to unaffected limbs)	1.36 ± 0.23
LLIS score, mean ± SD	
Physical concerns	23.9 ± 7.4
Psychosocial concerns	12.5 ± 3.3
Functional concerns	17.9 ± 5.4
Overall score	54.3 ± 15.9

SD, standard deviation; LLIS, Lymphedema life impact scale.

**Table 3:** Single-photon emission computed tomography analysis.

Result	N
Negative finding (Neither pelvic retention nor retrograde lymphatic flow)	3 (30.0%)
Pelvic retention only	1 (10.0%)
Lower limb retrograde lymphatic flow only	0
Pelvic retention with lower limb retrograde lymphatic flow	6 (60.0%)
Extent of lower limb retrograde lymphatic flow	
Thigh	6 out of 6 (100%)

N, number.

**Table 4:** Comparison of the reverse (R) group versus non-reverse (NR) group.

Characteristics	R	NR	*p*-Value
Circumference ratio (affected to unaffected limbs), mean ± SD			
Upper thigh (point 1)	1.14 ± 0.10	1.11 ± 0.07	0.796
Midthigh (point 2)	1.17 ± 0.12	1.21 ± 0.10	0.587
Superior border of patella (point 3)	1.13 ± 0.08	1.20 ± 0.14	0.269
Inferior border of patella (point 4)	1.17 ± 0.14	1.25 ± 0.04	0.089
Midcalf (point 5)	1.17 ± 0.12	1.16 ± 0.04	0.181
Ankle (point 6)	1.17 ± 0.17	1.24 ± 0.17	0.941
Mean volume ratio (affected to unaffected limbs)	1.33 ± 0.26	1.42 ± 0.16	0.374
LLIS score, mean ± SD			
Physical concerns	24.0 ± 7.4	23.6 ± 8.9	0.728
Psychosocial concerns	12.7 ± 3.1	12.0 ± 4.3	0.475
Functional concerns	18.1 ± 5.8	17.3 ± 5.1	0.631
Overall score	54.8 ± 16.3	53 ± 18.1	0.857

SD, standard deviation; LLIS, Lymphedema life impact scale.

## Data Availability

The raw data supporting the conclusions of this article will be made available by the authors on an appropriate request.
